# Optical Properties of Titanium in the Regime of the Limited Light Penetration

**DOI:** 10.3390/ma13040952

**Published:** 2020-02-20

**Authors:** Lukasz Skowronski

**Affiliations:** Institute of Mathematics and Physics, UTP University of Science and Technology, Kaliskiego 7, 85-796 Bydgoszcz, Poland; lukasz.skowronski@utp.edu.pl

**Keywords:** magentron sputtering, titanium, optical properties, resistivity, penetration depth

## Abstract

In this study, the titanium layers from 12 to 1470 nm thick were fabricated by using the method involving dynamically changed working gas pressure (gas injection magnetron sputtering). The influence of the deposition time on the optical and electrical properties of Ti films, as well as on their microstructure, are considered. The samples are investigated by means of spectroscopic ellipsometry, atomic force microscopy, X-ray diffraction, and confocal optical microscopy. Additionally, for the Ti layers, the sheet resistance was determined. The produced coatings exhibit privileged direction of growth (002). The obtained results show a gradual increase in the mean relaxation time of free-carriers with the increase in the thickness of titanium film. However, the plasma energy exhibits maximum for the coating with the thickness of 93 nm. For such thickness, the lowest value of optical resistivity (about 200 μΩ cm) was observed. It was found that the dc- and optical resistivity exhibit similar values for titanium films with thickness up to 93 nm. For thicker Ti layers, significant differences in resistivities (dc- and optical) were noticed. The behavior of the Drude parameter (the plasma energy), calculated optical resistivity, and discrepancies between values of optical and dc-resistivities for thicker Ti coatings can be explained as a result of the limited light penetration.

## 1. Introduction

The microstructural and optical properties of the materials synthesized in the vacuum chamber strongly depend on the growing conditions (e.g., a pressure of working/reactive gas, deposition rate, substrate temperature, and the bias applied) [1]. There are various methods used to increase energy of condensed particles, e.g., heating of the substrate (and/or post-deposition annealing) [2,3], plasma bombardment [4], increasing of the power of the supplier [3,5]. Sometimes the additional voltage, the bias, is applied during the coating formation process [4,6,7]. Those methods lead to increasing the kinetic energy of particles dissipated due to the inter-particle collisions. The above mentioned problem is very important since the microstructure and phase composition of nitrides/oxides strongly depend on the energy of condensed particles and the effect of macroscopic properties of coatings; hardness, corrosion resistance, and optical properties. Moreover, as shown in Reference [8], the energy conditions during the layer formation are associated only with condensable particles if the temperature of substrate is lower than 180 ${}^{\circ }$C.

A completely different approach is applied in gas injection magnetron sputtering (GIMS) technique [9,10,11]; the variable pressure of plasma forming gas is used to control the plasma process. The impulse injection of a working gas into the vacuum chamber elongates the mean free path of gas phase particles and decreases inter-particles’ collisions probability. This means that the kinetic energy of gas phase particles is practically not dissipated before their condensation on the substrate. The GIMS method was successfully used to produce decorative multilayers [12,13,14,15,16]. The recent investigation clearly shows that applying the GIMS technique allows for synthesizing the nanocrystalline rutile phase of TiO${}_{2}$ directly during the deposition on the unheated substrate [17].

The aim of this work is to examine the influence of the thickness of titanium films (from 12 nm to 1470 nm) formed using the GIMS method on their microstructure, optical and electrical properties. It should be noted that microstructural properties of Ti coatings prepared under different conditions and using various modifications of magnetron sputtering methods are described in literature [2,3,4,5,6,7,18,19,20,21]; however, their optical constants and/or opto-electronic properties are described only in a few publications [20,22,23,24]. Additionally, the results related to the optical constants of Ti can b found in Reference [12,14,15]. It should be noted that the optical properties (the complex dielectric function, reflectance and transmittance spectra) of semitransparent titanium coatings (the thickness of a layer below 50 nm) were described in Reference [13,14]. However the description was limited and related to the influence of the complex dielectric function of Ti deposited on glass on reflectance and/or transmittance spectra of Ti/glass Ti/Al systems and then on the color formation (and transparency) of TiO${}_{2}$/Ti/glass and TiO${}_{2}$/Ti/Al structures. Some of the results included in Reference [13] are presented in this publication as a part of a larger research, related to the systematic investigation on influence of the thickness (two orders of magnitude) of titanium coatings on their properties, and are precisely marked in the presented manuscript.

In general, the resistivity of metallic layer deposited at established growth conditions decreases with the increase in the thickness. In the presented manuscript it was shown that the behavior of the optical resistivity may be reverse when the metallic coating is thick enough, while the dc-resistivity keeps the above-mentioned trend. This effect has been explained as a result of the limited penetration of light (the optical response is related only to a top part of the Ti coating).

## 2. Materials and Methods

### 2.1. Sample Preparation

The Ti layers were deposited on Bk7 glass plates (1 mm thick) using the GIMS method applying the industrial magnetron line in the Bohamet company [25]. Before the coating production the substrate was cleaned in a solution based on a mixture of alcohols and surfactants. Moreover, the plasma cleaning of the glass was performed before the deposition of titanium coatings.

To deposit titanium coatings, two linear Ti targets (grade 1), 2500 × 100 × 10 mm, were used. The target-substrate distance was 140 mm. The DPS-type power supplier (Dora Power Systems) [26] was used during the deposition process. The power supply was operated in 100 kHz sinusoidal pulses grouped in the 2 kHz unipolar pulse packets. The detailed description of the GIMS method therein voltage characteristics can be found in Reference [9,17,27,28,29].

The pressure in the vacuum chamber before the deposition was $p_{0}<$ 0.01 Pa, while, during the deposition, the average pressure was *p* = 0.06 Pa. The sputter gas (argon; 5N) was injected as 3 Hz impulses [9,17]. The Ti layers with the various thickness were produced by changing the deposition time from 11 s to 2240 s.

### 2.2. Sample Characterization

The Atomic Force Microscope (AFM) Innova (Bruker) device (with the standard Si tips for a tapping mode) was used to examine the surface topography of the Ti films. The scan size was 2 μm × 2 μm. The roughness parameter $R_{a}$ (the arithmetical mean deviation of the assessed profile) was determined using the NanoScope Analysis software (version 1.40). The $R_{a}$ quantity is defined as:

(1) $$R_{a}=\frac{1}{N}\sum_{j=1}^{N}\left|Z_{j}\right|.$$

In Equation (1), $Z_{j}$ is the current surface height value, while *N* is the number of points measured.

The XRD measurements were performed using PANanalytical X-Pert PRO X-Ray diffractometer with Ni-filtered Cu K${}_{\alpha }$ radiation (λ = 1.5418 Å).

A sheet resistance (4PP) was measured with a four-point probe (from Jandel Engineering Ltd.) with the Keithley nanovoltmeter. The measurements were performed at several points on the surface of a titanium film.

Ellipsometric azimuths Ψ and Δ were measured for two or three angles of incidence (65${}^{\circ }$ and 75${}^{\circ }$ for non-transparent or 65${}^{\circ }$, 70${}^{\circ }$, and 75${}^{\circ }$ for semitransparent samples) in the NIR-vis-UV spectral range (193–2000 nm; 0.6–6.5 eV) by the V-VASE (from J.A.Woollam Co., Inc.) device. Additionally, for semitransparent samples the transmittance measurements for near-normal incidence were performed by means of the Cary 5000 spectrophotometer. The WVASE32 software (J. A. Woollam Co., Inc.) was used to perform the fit procedure. The spectroscopic ellipsometry (SE) technique, combined with transmittance measurements, was used to determine thicknesses of the rough layer and the titanium film (only semitransparent samples), as well as optical constants of the metallic coatings.

The thickness of the opaque titanium films was estimated from a linear interface layer/substrate profile using the confocal optical microscope (COM) Lext OLS 4000 (from Olympus). Those measurements were performed at ten points on the profile.

## 3. Results and Discussion

The thickness of titanium coatings ($d_{Ti}$) was established using the spectroscopic ellipsometry technique and the confocal optical microscope. Detailed description related to the determining of the thickness of Ti will be presented in the ’Optical and Electrical Properties of the Titanium Coatings’ section; however, in the ’Results and Discussion’ section, these thicknesses will be used as a part of the name of a sample (in the following form: Ti($d_{Ti}$ rounded to 1 nm)) and during discussion of the obtained results. Due to a strong dependence of microstructural, electrical and optical properties on the metallic layer thickness [30], the obtained results are discussed as a function of $d_{Ti}$.

### 3.1. Topography of Ti Coatings

**Figure 1:**
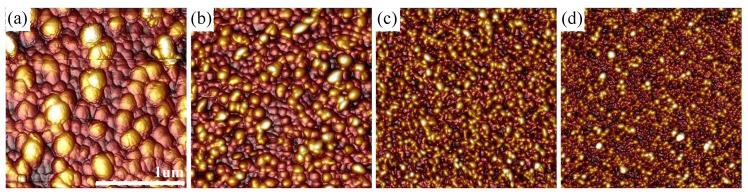
Atomic Force Microscope (AFM) images (2 μm × 2 μm) of Ti layer surfaces for: (**a**) Ti(1470), (**b**) Ti(743), (**c**) Ti(363), and (**d**) Ti(93) samples.

Figure 1 presents the surface topography of titanium coatings for different thickness. The AFM images for the thinner Ti films were presented in Reference [13]. One can see that these topographies show significant dependence on the time of deposition ($t_{Ti}$) and thereby on the thickness of the Ti film, especially for thicknesses larger than 50 nm. The lateral grain size increases with $d_{Ti}$ from about 50–100 nm for the Ti(12) sample to 100–300 nm for the Ti(1470) specimen. The values of $R_{a}$ for the produced metallic films (see Table 1) are in the range from about 2 nm (for Ti(12)) to about 8 nm (for Ti(1470)). For the thicker titanium coatings the values of $R_{a}$ are negligibly low in comparison with the coating thickness. This result indicate that the obtained coatings are relatively smooth.

### 3.2. XRD Measurements

**Figure 2:**
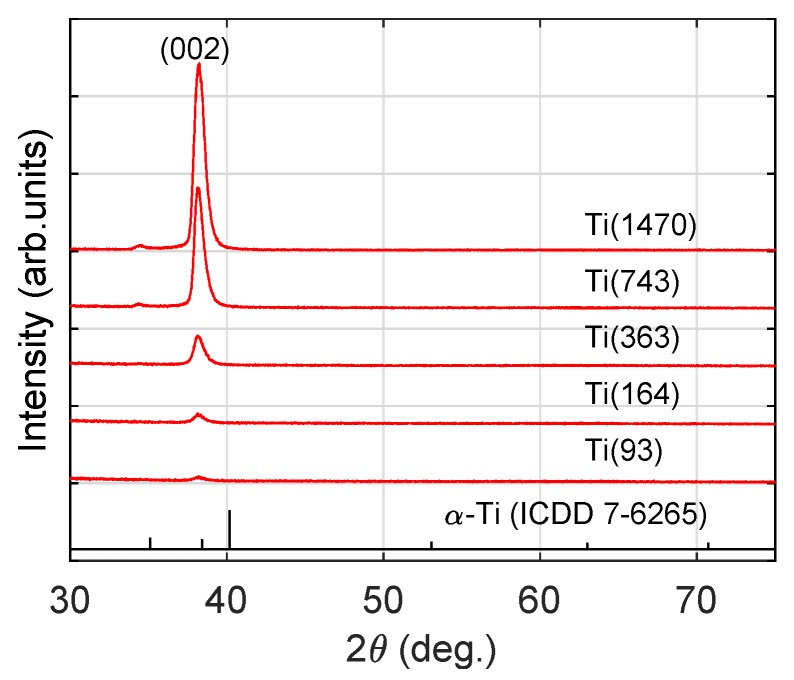
Diffractograms of the Ti layers.

The XRD pattens recorded for the produced Ti coatings are presented in Figure 2. According to the #ICSD 7-6265 card, α-Ti (the hexagonal-close-packed form (hcp); latice constants: *a* = 2.950 Å, *c* = 4.685 Å) has three main diffraction peaks in the 2θ range from 35${}^{\circ }$ to 41${}^{\circ }$ (calculated for Cu K${}_{\alpha }$ radiation) and three, less intensive, for 50${}^{\circ }<2\theta <$ 70${}^{\circ }$. The primary diffraction peaks at 2θ = 35.1${}^{\circ }$, 38.2${}^{\circ }$ and 40.2${}^{\circ }$ correspond to reflections from (100), (002) and (101) planes, respectively. XRD patterns presented in Figure 2 exhibit only one peak, which could be attributed to reflections from planes with Miller indices (002). No signals at 2θ = 35.1${}^{\circ }$ and 40.2${}^{\circ }$ were detected. A week signal recorded at 2θ = 34.5${}^{\circ }$ is associated with unfiltered Cu K${}_{\beta }$ radiation and is visible in XRD patterns for Ti(743) and Ti(1470) samples due to a high intensity of the (002) peak. This indicates the strong texturization of coatings produced applying the GIMS method. Similar results, with only one peak from (002) planes, were previously obtained by Jung [6] for the Ti layers produced using grid-attached magnetron and relatively high value of the bias polarization (−150 V), as well as by Chawla [3] for the Ti coatings formed using a relatively high power density. Despite the fact that (002) orientation is preferred for titanium layers deposited on glass [20], the results indicate that only one growth direction is characteristic for high-energy condensed particles.

The average size of nanocrystallites was estimated using the Scherrer formula:

(2) $$<D>=\frac{0.9\lambda }{\beta cos\left(2\theta \right)}.$$

In Equation (2), λ is the X-ray wavelength (λ=1.5418 Å), and β is the full-width at half-maximum (FWHM) of the Bragg diffraction peak at angle 2θ. The estimated grain size <D> is 7–10 nm with uncertainty at the level of 3–5 nm (see Table 1) and does not show the thickness of coating dependence.

### 3.3. Optical and Electrical Properties of the Titanium Films

**Figure 3:**
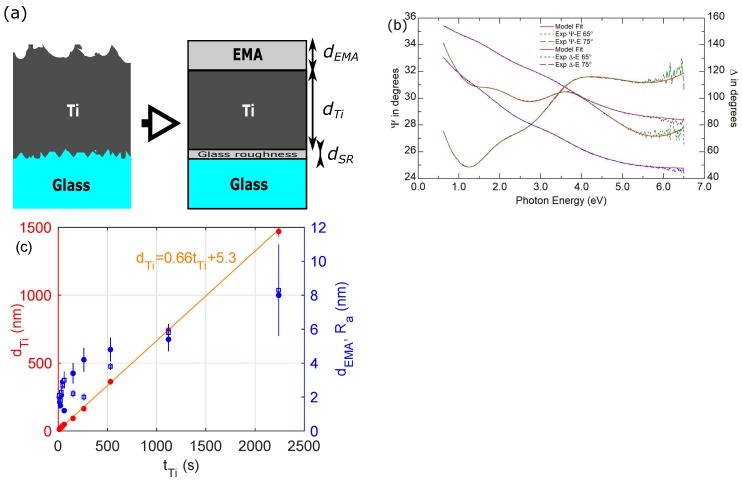
(**a**) A structure of the Ti coating deposited on the glass substrate and an optical model of a sample ($d_{EMA}$ - the thickness of the rough layer, $d_{Ti}$ - the thickness of the titanium layer). (**b**) Experimental Ψ and Δ azimuths for two angles of incidence (65${}^{\circ }$ and 75${}^{\circ }$) and their model fits for the Ti(363) sample. The fit was performed using *P* = 12 parameters ($\chi^{2}$ = 1.6). (**c**) Thicknesses of the titanium coating ($d_{Ti}$) and the rough layer ($d_{EMA}$; filled circles), as well as value of the $R_{a}$ parameter (open squares). The solid line represents the linear fit of $d_{Ti}$ versus $t_{Ti}$ dependence. EMA = Effective Medium Approximation.

Figure 3a shows a structure of titanium coating deposited on the glass substrate. To determine the complex dielectric function of titanium coatings, as well as the thickness of the Ti films, the five-phase (or three-phase—for thicker layers) optical model of a sample was used: ambient/rough layer/Ti layer/intermix layer/Bk7 glass (see Figure 3a). For thicker layers (samples with $d_{Ti}\geq$ 93 nm), the glass substrate and the intermix layer were omitted during the analysis of spectroscopic ellipsometry results.

To describe optical constants of the rough layer, the two ($d_{Ti}\leq$ 49 nm) or three (for $d_{Ti}\geq$ 93 nm) component Bruggeman Effective Medium Approximation (EMA) [31,32] model was used as a combination of optical properties of native titanium dioxide and titanium or void, native titanium dioxide and titanium (the assumed volume fraction of each medium was 1/2—for the two component structure or 1/3 for the three component system). This approach to specification of the structure and optical properties of the rough layer for various thicknesses of titanium coatings was made for a more detailed description of the produced coatings. The optical constants of native TiO${}_{2}$ were taken from the J.A. Woollam optical constants database [32]. The non-ideal glass-titanium layer was also described using the Bruggeman Effective Medium Approximation model with fractions of glass and titanium (set at 1/2). Its thickness was established to be 2 nm. The optical constants of glass substrate were established in the separate experiment [12,13].

The Drude-Lorentz model was used to parameterize the complex dielectric function ($\tilde{\varepsilon }$) of titanium films [31,32]:

(3) $$\tilde{\varepsilon }=\varepsilon_{\infty }-\frac{{\left(\hbar \omega_{p}\right)}^{2}}{E^{2}+\left(\hbar \Gamma \right)E}+\sum_{j=1}^{n}\frac{A_{j}E_{j}^{2}}{E_{j}^{2}-E^{2}-iBr_{j}E}.$$

In Equation (3), $\varepsilon_{\infty }$ is the high-frequency dielectric constant (set as 1), $\hbar \omega_{p}=E_{p}$, and $\hbar \Gamma =E_{\Gamma }$ are Drude parameters (the unscreened plasma frequency and the free-carrier damping, respectively), while $A_{j}$, $E_{j}$, and $Br_{j}$ are quantities describing the Lorentzian oscillator (the amplitude, energy, and broadening of the *j*-th absorption band, respectively). The model quantities were varied to minimize the reduced mean squared error, $\chi^{2}$, defined as [32]:

(4) $$\chi^{2}=\frac{1}{N-P}\sum_{j}\left(\frac{{\left(\Psi_{j}^{mod}-\Psi_{j}^{exp}\right)}^{2}}{\sigma_{\Psi \;j}^{2}}+\frac{{\left(\Delta_{j}^{mod}-\Delta_{j}^{exp}\right)}^{2}}{\sigma_{\Delta \;j}^{2}}\right).$$

In Equation (4), *N* is the total number of data points, and *P* is the number of fitted model parameters. The $\Psi_{j}^{exp}$ and $\Delta_{j}^{exp}$ are experimental ellipsometric azimuths. The quantities with superscript ’mod’ correspond to the calculated Ψ and Δ azimuths. The sigma is a standard deviation determined for Ψ ($\sigma_{\Psi \;j}$) and Δ ($\sigma_{\Delta \;j}$) quantities. An example of the fit is presented in Figure 3b. The determined values of $\chi^{2}$ are lower than 1.6 for all the samples examined in this study.

The estimated thickness of titanium film ($d_{Ti}$) and the rough layer ($d_{EMA}$) are summarized in Table 1. Figure 3c presents the thickness ($d_{Ti}$) of titanium coatings as a function of the deposition time ($t_{Ti}$). The estimated $d_{Ti}$ values for Ti layers fabricated using the GIMS method fall within the range from 12 to 1470 nm. Linear dependence $d_{Ti}\left(t_{Ti}\right)=0.66t_{Ti}+5.3$ (see Figure 3) allows to estimate the deposition rate ($d_{r}$) of Ti layers which was established to be 40 nm/min. The $d_{EMA}$ values are in the range from 1 to 8 nm and coincide well with the $R_{a}$ values determined from the AFM measurements (see Table 1).

**Figure 4:**
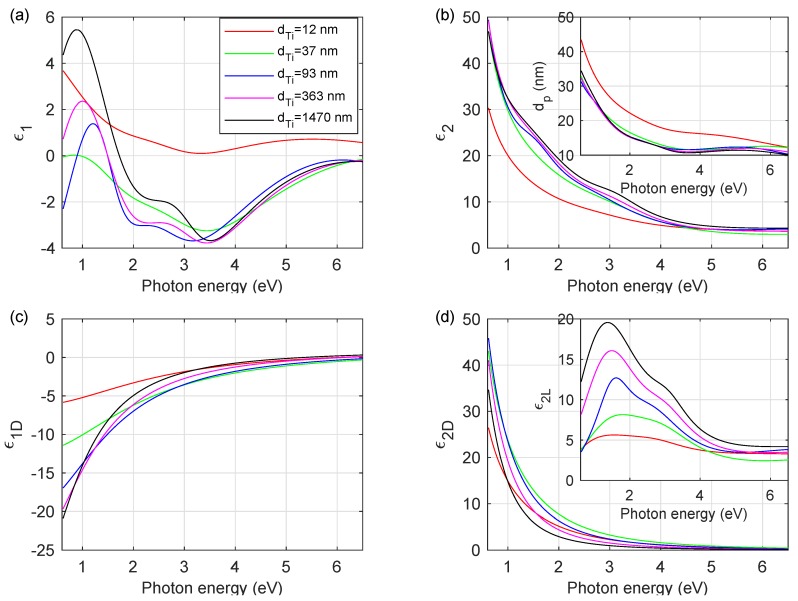
(**a**) The real ($\varepsilon_{1}$) and (**b**) imaginary ($\varepsilon_{2}$) parts of the complex dielectric function ($\tilde{\varepsilon }$) of the titanium films. Inset: the penetration depth ($d_{p}$). (**c**) The real ($\varepsilon_{1D}$) and (**d**) imaginary ($\varepsilon_{2D}$) parts of the Drude contribution to $\tilde{\varepsilon }$. Inset: The separate contributions from the Lorentz terms ($\varepsilon_{2L}$) to the imaginary part of $\tilde{\varepsilon }$. The complex dielectric function of the Ti films of thickness equals 12 nm and 37 nm is taken from Reference [13].

Figure 4a,b show the real ($\varepsilon_{1}$) and imaginary ($\varepsilon_{2}$) parts of the complex dielectric function of Ti coatings, respectively. To make the figure (Figure 4) more clear, the $\tilde{\varepsilon }$ curves for chosen thicknesses of titanium layers are drawn. The complex dielectric functions for the other produced Ti coatings are located between the curves presented in Figure 4. The differences in $\varepsilon_{1}$ and $\varepsilon_{2}$ of particular films are visible in the NIR-vis spectral range. This effect has two sources: the change of Drude contribution to the complex dielectric function (see Figure 4c,d) and, more evident, an increase in maximum in $\varepsilon_{1}$ spectra near 1.0 eV with an increase in the Ti layer thickness. The $\tilde{\varepsilon }$ demonstrates typical features for Ti metallic samples: the Drude term in the IR spectral range and interband transition at 3.0–3.3 eV. In the inset of Figure 4d the Lorentzian contribution ($\varepsilon_{2L}$) to the complex dielectric function is presented. The shape of $\varepsilon_{2L}$ exhibits two maximums. The first absorption feature, located at about 3 eV, is associated with the interband transitions of Ti. The second one (at about 1.0–1.5 eV) slightly shifts toward longer wavelengths with an increase in titanium layer thickness and is related to the granular structure of the titanium films. Most probably, these resonance features are related to plasmonic effect associated with a granular structure of Ti films and was observed earlier for pure metals [33] and alloys [34,35].

To obtain detailed description of absorption features related to the free-electrons, the Drude parameters ($\hbar \omega_{p}$ and ℏΓ) were determined during the analysis of spectroscopic ellipsometry results. The estimated values of $\hbar \omega_{p}$ and ℏΓ are summarized in Table 2. The Drude parameters $\hbar \omega_{p}$ and ℏΓ are associated with the concentration and scattering frequency of free electrons, respectively. Based on their values, two additional parameters describing the free-carriers can be calculated. The mean relaxation time of conduction electrons is defined as:

(5) $$\tau =\Gamma^{-1},$$

while the optical resistivity at ℏω=0 (corresponding to the dc-resistivity):

(6) $$\rho_{o}=\frac{\hbar \Gamma }{{\left(\hbar \omega_{p}\right)}^{2}}.$$

**Figure 5:**
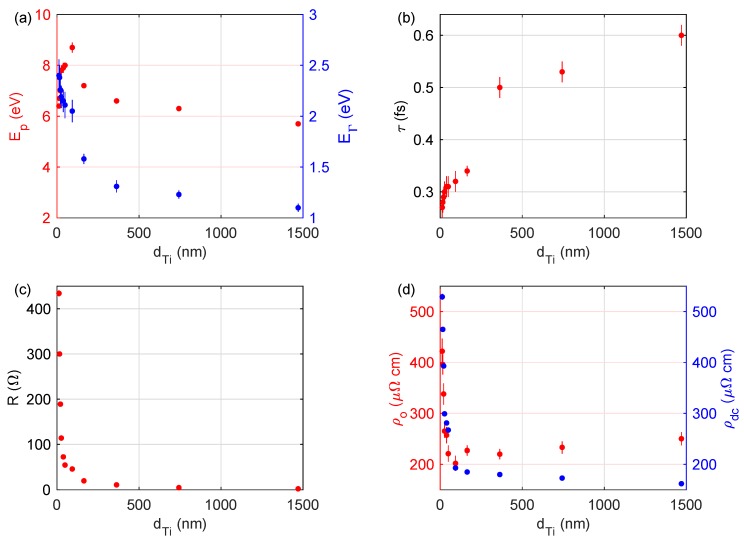
(**a**) The plasma energy ($E_{p}=\hbar \omega_{p}$) and the free-carrier dumping ($E_{\Gamma }=\hbar \Gamma$), (**b**) the mean relaxation time of conduction electrons (τ), (**c**) the sheet resistance (*R*), and (**d**) the optical ($\rho_{o}$) and dc-resistivity ($\rho_{dc}$) determined for the examined Ti coatings plotted as a function of the thickness of the Ti film.

The determined values of $\hbar \omega_{p}$, ℏΓ, τ and $\rho_{o}$ for the Ti coatings are summarized in Table 2. In order to observe more easily the trends in changes of $\hbar \omega_{p}$, ℏΓ, τ and $\rho_{o}$ parameters, their values have been plotted (Figure 5) as a function of the thickness of titanium coating. Figure 5a,b present the plasma energy ($\hbar \omega_{p}=E_{p}$), the free carrier dumping ($\hbar \Gamma =E_{\Gamma }$), as well as the mean relaxation time of conduction electrons (τ), respectively. The $\hbar \omega_{p}$ value increases from 6.4 eV to 8.7 eV with the increase in the thickness of the titanium coating from 12 to 93 nm and then decreases from 8.7 eV to 5.7 eV for larger values of $d_{Ti}$. The mean relaxation time of conduction electrons (τ) increases from 0.27 eV (for the Ti(12) sample) to 0.60 eV (for the Ti(1470) sample). It should be noted that, for most metallic coatings deposited at established growth conditions (only time of deposition is varied, thus varying the thickness of a layer), the influence of the thickness of a layer on the value of τ (and Γ) shows the tendency presented in Figure 5a,b; however, the determined $\hbar \omega_{p}\left(d_{Ti}\right)$ relation exhibits the pronounced maximum ($\hbar \omega_{p}$(93 nm) = 8.7 eV). Both $\hbar \omega_{p}$, as well as τ, directly affect the $\rho_{o}$ value (Equations (5) and (6)). The $\rho_{o}$ for the thinnest Ti coating was established to be 422 μΩcm; for the thickest, it was 250 μΩcm. However, the minimum value of $\rho_{o}$ = 202 eV was obtained for the sample with $d_{Ti}$ = 93 nm, that is, for the coating with the highest value of $\hbar \omega_{p}$.

To explain the unexpected tendency in $Ep\left(d_{Ti}\right)$ and $\rho_{o}\left(d_{Ti}\right)$ (the existence of extrema) the 4PP measurements were performed for all the samples prepared. A sheet resistance (*R*) values are in the range from 434 Ω for the Ti(12) sample to about 2 Ω for the Ti(1470) specimen and are summarized in Table 2. The sheet resistance of the Ti coatings demonstrates behavior typical for metals; *R* value decreases with $d_{Ti}$ increasing, which is presented in Figure 5c. The dc-resistivity of the produced titanium coatings ($\rho_{dc}=R\;d_{Ti}$) decreases from 529 μΩ cm to 162 μΩ cm with the increase in the thickness of the Ti layer from 12 to 1470 nm (see Table 2). For semitransparent titanium coatings ($d_{Ti}\leq 49$ nm) [13] the $\rho_{dc}$ values are higher than adequate $\rho_{o}$ ones. The discrepancies are lower than 5% for the titanium films with $d_{Ti}$=93 nm and are about 20% for the Ti layer with $d_{Ti}$ = 12 nm. This agreement is satisfactory, considering a completely different experimental methods used to determine $\rho_{o}$ and $\rho_{dc}$ quantities. For the thicker Ti layers ($d_{Ti}\geq$ 93 nm) the value of $\rho_{dc}$ still decreases with increase in the $d_{Ti}$. It should be noted that apart from the quite significant percentage difference between $\rho_{o}$ and $\rho_{dc}$ (up to 40%), the behavior of $\rho_{dc}$ is opposite to the behavior of $\rho_{o}$ (i.e., the decrease in $\rho_{dc}$ with the increase in $d_{Ti}$).

According to the hypothesis based on the Matthiesen’s rule, the electrical resistivity ($\rho_{dc}$) of a non-ideal metal film can be expressed as:

(7) $$\rho_{dc}=\rho_{dc0}+\rho_{gb}+\rho_{ss}+\rho_{sr},$$

where $\rho_{dc0}$ is the residual resistivity, and $\rho_{gb}$, $\rho_{ss}$ and $\rho_{sr}$ are contribution from grain-boundary, surface and surface roughness scattering, respectively. For thinner coatings the main factors influence the resistivity are related to the film-substrate and film-ambient interfaces, as well as the surface roughness. For thicker layers the thickness of a film (and in the presented study limited surface roughness) is less evident. The coatings with larger grains (thicker layers) contain relatively a few grain boundaries and this effect plays a major role in increasing of the mean relaxation time of conduction electrons, whereas the plasma energy decreasing with an increase in the layer thickness is associated with the density of the material. For thicker films the subsurface structure is less packed (see Figure 1). The presented clarification explains correctly the typical behavior of $\rho_{dc}$ for 12 nm$\leq d_{Ti}\leq$ 1470 nm and $\rho_{o}$ for $d_{Ti}\leq$ 93 nm (see Figure 5d); however, the behavior of $\rho_{o}$ for the thicker titanium coatings ($d_{Ti}>$ 93 nm) is still unexplained. The discrepancies between $\rho_{o}$ and $\rho_{dc}$ values for thicker titanium coatings can be explained as an effect of limited light penetration. Figure 4b (the inset) shows the penetration depth [32]:

(8) $$d_{p}=\frac{\lambda }{4\pi k}$$

of light as a function of photon energy. In Equation (8), λ is a wavelength, and *k* is the extinction coefficient ($k=Im\left(\sqrt{\tilde{\varepsilon }}\right)$). This quantity informs about the depth from the surface where the light intensity decreases *e*-times. The maximum $d_{p}$ value falls in the NIR spectral range and does not exceed 45 nm for the thinnest layer and it is lower than 35 nm for the thicker Ti coatings. In the visible and ultraviolet spectral range the $d_{p}$ value is lower than 15 nm (20 nm for the thinner Ti film). However, if the layer thickness is higher than the $d_{p}$ value, determining its thickness is still possible. Spectroscopic ellipsometry has a very high precision up to thickness ∼5 d${}_{p}$ [31,36]. Therefore, in an optical model for thinner samples ($d_{Ti}\leq$ 49 nm), the substrate (Bk7 glass) was considered. The thicknesses of the other titanium films examined are larger (except the Ti(93) sample) than ∼5 d${}_{p}$ (see Table 1) and, consequently, during the SE measurement, the optical response to incident electromagnetic radiation was recorded only from a top part of the Ti coating. Therefore, the determined complex dielectric function and parameters describing opto-electronic properties of layers ($\hbar \omega_{p}$, ℏΓ, τ and $\rho_{o}$) do not represent whole coating; however, they mainly contain information about their subsurface structure. Due to the relatively high thickness of the titanium coatings investigated ($d_{Ti}>$ 93 nm) and the limited penetration depth of the electromagnetic radiation into the layer, the opto-electronic properties of Ti films should be discussed taking this fact into account. These facts explain the behavior of the plasma energy ($\hbar \omega_{p}$) and the optical resistivity ($\rho_{o}$) and thus discrepancies between the optical and dc-resistivities for the thicker titanium films ($d_{Ti}>$ 93 nm).

## 4. Conclusions

The optical, electrical, and microstructural properties of titanium coatings of thickness from 12 to 1470 nm produced using the GIMS technique were examined by means of atomic force microscopy, X-ray diffraction, spectroscopic ellipsometry, confocal optical microscopy, and a four-point probe techniques. The prepared layers exhibit granular structure, which affects their optical properties. The Ti films show the privileged direction of growth (002). The average size of nanocrystallites was established to be 7–10 nm. The complex dielectric function of Ti films is typical for conducting materials: the free-carrier absorption, interband transitions, and additional absorption related to the granular structure of a coating. The free-carrier dumping (ℏΓ) and the mean relaxation time of conduction electrons (τ) exhibit a typical behavior for metallic coatings; however, the plasma energy ($\hbar \omega_{p}$) and the optical resistivity ($\rho_{o}$) show extreme behavior, which can be ascribed to the granular structure of the top part of a Ti coating. The differences between optical ($\rho_{o}$) and dc-resistivities ($\rho_{dc}$) can be explained by the limited penetration depth of light, much less in comparison with the thickest films.

## Figures and Tables

**Table 1:** Drude parameters ($\hbar \omega_{p}$ and ℏΓ), the mean relaxation time of conduction electrons (τ), the dc-resistivity ($\rho_{dc}$), the optical resistivity ($\rho_{o}$), and the sheet resistance (*R*) of the Ti layers.

Sample	$\hbar \omega_{p}$ (eV)	ℏΓ (eV)	τ (fs)	$\rho_{o}$(μΩ cm)	$\rho_{\mathrm{dc}}$(μΩ cm)	*R*(Ω)
Ti(12) *	6.4 ± 0.1	2.40 ± 0.16	0.27 ± 0.02	422 ± 25	529 ± 6	434 ± 7
Ti(16) *	6.7 ± 0.1	2.38 ± 0.12	0.28 ± 0.02	396 ± 20	465 ± 5	300 ± 5
Ti(21) *	7.0 ± 0.1	2.26 ± 0.14	0.29 ± 0.02	338 ± 21	393 ± 4	189 ± 3
Ti(26) *	7.8 ± 0.2	2.19 ± 0.11	0.30 ± 0.02	265 ± 13	299 ± 3	114 ± 2
Ti(37) *	7.9 ± 0.2	2.15 ± 0.11	0.31 ± 0.02	257 ± 16	281 ± 1	72.8 ± 0.6
Ti(49) *	8.0 ± 0.1	2.11 ± 0.13	0.31 ± 0.02	221 ± 16	267 ± 1	54.7 ± 0.5
Ti(93)	8.7 ± 0.2	2.05 ± 0.11	0.32 ± 0.02	202 ± 15	193 ± 1	46.0 ± 0.1
Ti(164)	7.2 ± 0.1	1.58 ± 0.05	0.42 ± 0.01	227 ± 10	185 ± 1	19.8 ± 0.2
Ti(363)	6.6 ± 0.1	1.31 ± 0.06	0.50 ± 0.02	220 ± 10	180 ± 1	10.9 ± 0.2
Ti(743)	6.3 ± 0.1	1.23 ± 0.04	0.53 ± 0.02	233 ± 12	173 ± 1	4.76 ± 0.06
Ti(1470)	5.7 ± 0.1	1.10 ± 0.04	0.60 ± 0.02	250 ± 13	162 ± 1	2.18 ± 0.04

* values of $\hbar \omega_{p}$, ℏΓ and τ estimated for the examined Ti coatings are taken from Reference [13]; the values of $\rho_{dc}$ and *R* for these coatings were not published earlier.

**Table 2:** The time of deposition ($t_{Ti}$), the thickness of titanium film ($d_{Ti}$) and the EMA layer ($d_{EMA}$), the roughness parameter ($R_{a}$) and the average size of nanocrystallites (<D>).

Sample	$t_{\mathrm{Ti}}$ (s)	$d_{\mathrm{Ti}}$ (nm)	$d_{\mathrm{EMA}}$ (nm)	$R_{a}$ (nm)	<D> (nm)
Ti(12) *	11	12.2 ± 0.1 ${}^{a}$	2.0 ± 0.1	2.1 ± 0.2	-${}^{c}$
Ti(16) *	15	15.5 ± 0.2 ${}^{a}$	1.7 ± 0.1	1.8 ± 0.5	-${}^{c}$
Ti(21) *	22	20.8 ± 0.3 ${}^{a}$	1.5 ± 0.1	1.8 ± 0.3	-${}^{c}$
Ti(26) *	30	26.2 ± 0.1 ${}^{a}$	2.1 ± 0.1	2.3 ± 0.3	-${}^{c}$
Ti(37) *	45	38.6 ± 0.1 ${}^{a}$	2.9 ± 0.1	2.7 ± 0.2	-${}^{c}$
Ti(49) *	60	48.8 ± 0.2 ${}^{a}$	1.2 ± 0.1	3.0 ± 0.5	-${}^{c}$
Ti(93)	150	93 ± 7 ${}^{b}$	3.4 ± 0.6	2.2 ± 0.2	8 ± 5
Ti(164)	260	164 ± 10 ${}^{b}$	4.2 ± 0.7	2.0 ± 0.2	10 ± 4
Ti(363)	530	363 ± 22 ${}^{b}$	4.8 ± 0.7	3.8 ± 0.2	7 ± 3
Ti(743)	1120	743 ± 11 ${}^{b}$	5.4 ± 0.7	5.8 ± 0.5	7 ± 3
Ti(1470)	2240	1470 ± 40 ${}^{b}$	8.0 ± 1.4	8.3 ± 2.7	7 ± 3

* values of $d_{Ti}$, $d_{EMA}$ and $R_{a}$ estimated for the examined Ti coatings are taken from Reference [13]. ^a^ The thickness of Ti coating determined using spectroscopic ellipsometry. ^b^ The thickness of Ti coating determined using confocal optical microscope. ^c^ Diffractograms were not recorded.
